# Stapler access and visibility in the deep pelvis: A comparative human cadaver study between a computerized right angle linear cutter versus a curved cutting stapler

**DOI:** 10.1186/1750-1164-5-7

**Published:** 2011-08-27

**Authors:** Toyooki Sonoda, Juan Carlos Verdeja, David E Rivadeneira

**Affiliations:** 1 Department of Surgery, Weill Medical College of Cornell University, New York, NY, USA; 2 Department of Surgery, Baptist Health South Florida Hospital of Miami, Miami, FL, USA; 3 Department of Surgery, Saint Catherine of Siena Medical Center, Smithtown, NY, USA

## Abstract

**Purpose:**

Distal rectal stapling is often challenging because of limited space and visibility. We compared two stapling devices in the distal rectum in a cadaver study: the iDrive™ right angle linear cutter (RALC) (Covidien, New Haven, CT) and the CONTOUR^® ^curved cutter (CC) (Ethicon Endo-Surgery, Cincinnati, OH).

**Methods:**

Twelve male cadavers underwent pelvic dissection by 4 surgeons. After rectal mobilization as in a total mesorectal excision, the staplers were applied to the rectum as deep as possible in both the coronal and sagittal positions. The distance from the pelvic floor was measured for each application. A questionnaire rated the visibility and access of the stapling devices. Measurements were taken between pelvic landmarks to see what anatomic factors hinder the placement of a distal rectal stapler.

**Results:**

The median (range) distance of the stapler from the pelvic floor in the coronal position for the RALC was 1.0 cm (0-4.0) vs. 2.0 cm (0-5.0) for the CC, p = 0.003. In the sagittal position, the median distance was 1.6 cm (0-3.5) for the RALC and 3.3 cm (0-5.0) for the CC, p < 0.0001. The RALC scored better than the CC in respect to: 1. interference by the symphysis pubis, 2. number of stapler readjustments, 3. ease of placement in the pelvis, 4. impediment of visibility, 5. ability to hold and retain tissue, 6. visibility rating, and 7. access in the pelvis. A shorter distance between the tip of the coccyx and the pubic symphysis correlated with a longer distance of the stapler from the pelvic floor (p = 0.002).

**Conclusions:**

The RALC is superior to the CC in terms of access, visibility, and ease of placement in the deep pelvis. This could provide important clinical benefit to both patient and surgeon during difficult rectal surgery.

## Background

Oncologic outcomes after surgical treatment of rectal cancer have been improved by techniques such as the total mesorectal excision (TME) [1,2]. The division of the distal rectum with adequate tumor clearance is a critical step in a successful TME, and this is most commonly achieved using a stapling device. The ability to place a stapling device deep in the pelvis with good visualization could determine whether a sphincter-preserving operation is performed, or whether an abdominoperineal resection would be necessary. A distal resection margin of 2 cm has been generally accepted as oncologically sound [3,4], although recent studies suggest that a resection margin as short as 1 cm is safe, especially after neoadjuvant chemoradiotherapy [5-7]. Even this 1 cm distal margin, however, could be jeopardized by patient factors and ergonomically incorrect stapling devices. Appropriate distal rectal stapling is of vital importance since there is a correlation between close distal rectal margins and rectal cancer recurrence [8]. Furthermore, struggling to place a rectal stapler at the pelvic floor could lead to unwarranted trauma to a malignant tumor or even to perforation of the rectum, risking local tumor recurrence.

Surgeons specifically trained in deep pelvic surgery may deal with a close distal margin utilizing a hand-sewn coloanal anastomosis, where the rectum is amputated from the anus through a transanal approach, and then an anastomosis is established between the colon and anal canal using manually placed sutures. This approach remains the gold standard in cases of a threatened distal margin. In reality, however, surgeons are often not trained to perform this procedure or find it challenging. This point is illustrated in a study of a nationwide database in the US, where patients treated by surgeons with a high volume of rectal cancer surgery (≥ 10 per year) were five times more likely to undergo a sphincter-saving operation for rectal cancer compared with low volume surgeons (1-3 cases per year) [9]. A dependable, user-friendly stapler designed for distal rectal stapling could potentially have allowed more of these patients to undergo a restorative operation. A stapling device should not only be easy to apply, but must also be reliable since failure to form a proper staple line could lead to serious adverse outcomes such as an anastomotic leak.

Distal rectal stapling remains one of the most difficult challenges in surgery. The pelvis is restricted by its bony confines, and within this space exist other structures such as the bladder, prostate, uterus, and vagina. This complexity is further magnified in a narrow male pelvis. A proper stapling device must have a small profile, small enough to be passed around a bulky rectal tumor within a fixed space, yet be large enough to occlude the entire rectum and divide it accurately at a right angle. A stapler that reliably and uniformly reaches the most distal aspect of the rectum will have major impact on surgical outcome and surgeon satisfaction, reducing the level of frustration associated with a critical part of a difficult operation. Thus far, advancements in stapling technology have allowed for more sphincter-saving procedures to be performed for distal rectal tumors, but despite this, the current stapling tools remain far from perfect.

The CONTOUR^® ^curved cutter (CC) (Ethicon Endo-Surgery, Cincinnati, OH) is a single-patient-use stapler that was designed with a curved head that cuts and staples. The device delivers four staggered rows of titanium staples, with a knife between the second and third row. When applied, it occludes the tissue on both sides of a 40-mm transection. The CC is available with "green" and "blue" staple cartridges that compress tissue to approximately 2 mm and 1.5 mm, respectively. The iDrive™ powered handle (Covidien, New Haven, CT) utilizes a microprocessor-controlled hand-held unit that controls the functions of stapler closing, firing, and cutting with the push of a button. The iDrive™ powered handle is compatible with a low-profile 45-mm right angle linear cutter (RALC) single use reload, which delivers four staggered rows of titanium staples, with a knife between the second and third row of staples. When applied, it occludes the tissue on both sides of a 45-mm transection. The RALC single use reload attaches to the iDrive™ powered handle such that the jaws are perpendicular to the shaft of the handle. In addition, the surgeon has the ability to select whether to use the device in a "green" (compresses tissue to 2.0 mm) or "blue" mode (compresses tissue from 1.5 mm to 2.0 mm) without having to change the staple reload cartridge.

A comparative study of these two innovative stapling devices was undertaken in a human cadaver model to evaluate stapler access and visibility in the deep pelvis. Furthermore, we aimed to study what anatomic factors present the biggest impediments to distal rectal stapling using the current stapling devices.

## Methods

Twelve male cadavers underwent pelvic dissection by four surgeons (all certified by the American Board of Surgery, three with board specialty in Colon and Rectal Surgery). A low midline incision was created, and the rectum was mobilized circumferentially to the pelvic floor as in a TME.

The anatomic landmarks of each pelvis were carefully studied. Distances were measured and recorded between: 1. the symphysis pubis and umbilicus, 2. the right and left anterior superior iliac spines, 3. the symphysis pubis and the sacral promontory, 4. the pelvic floor and the sacral promontory, 5. the tip of the coccyx and the symphysis pubis, 6. the right and left pelvic sidewalls (i.e., transverse diameter of the pelvic inlet), and 7. the anal verge and the pelvic floor.

**Figure 1:**
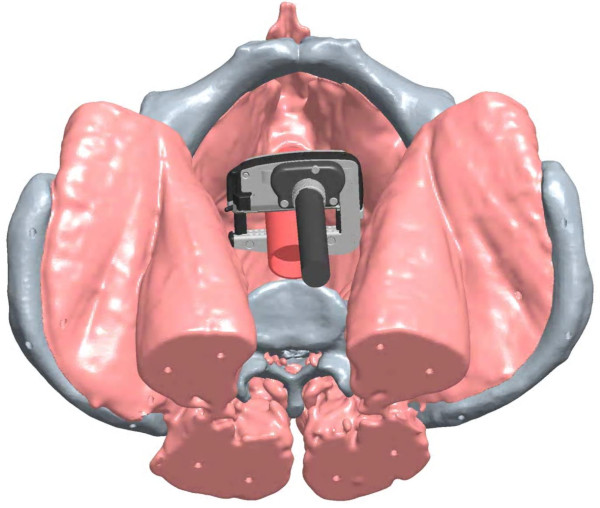
**Illustration of coronal placement of the RALC**.

**Figure 2:**
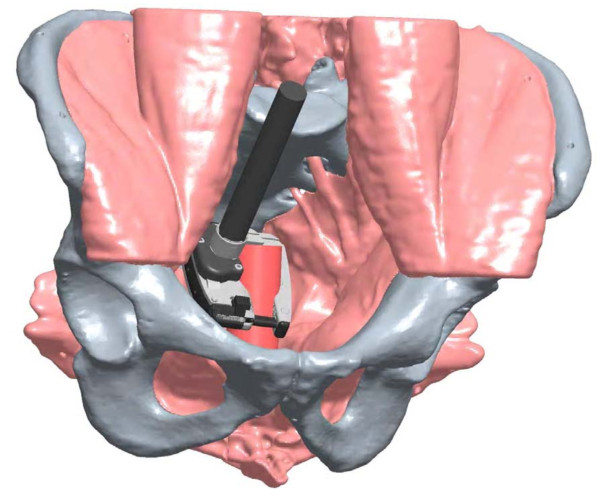
**Illustration of sagittal placement of the RALC**.

**Figure 3:**
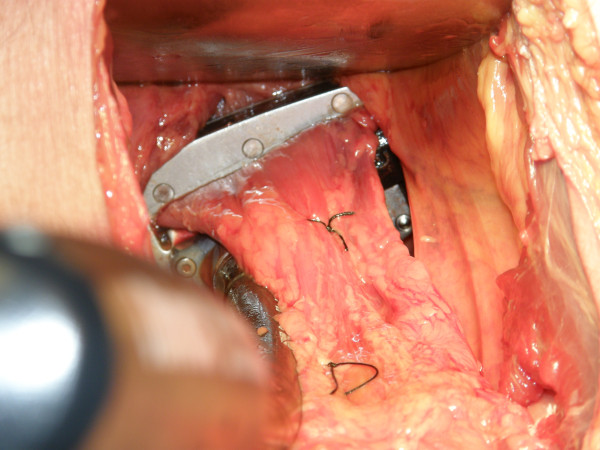
**Distal placement of the RALC on the rectum**.

Both the CC and RALC 45-mm stapling devices were applied to the rectum, advanced as deep as possible in the pelvis in both coronal and sagittal configurations (see Figure 1 and 2), and engaged. All four surgeons operated independently on all twelve cadavers, for a total of 48 applications for each stapler per position. To increase objectivity, randomization was used to determine which stapling device was used before the other. After the staplers were placed as distally as possible on the rectum (see Figure 3 for RALC depiction), the distance of the stapling device from the pelvic floor was measured. This was calculated from an initial suture that was placed on the anterior rectum 5 cm from the pelvic floor, prior to any engagement of stapling devices. The surgeons were then asked to answer a questionnaire that rated various aspects of access, visibility, and ease of placement of the stapling devices.

### Statistical Methods

To account for potential investigator and cadaver differences, regression models were used for statistical analysis. Linear regression was performed for continuous outcomes and logistic regression for binary and ordinal outcomes. Other than the main variable of interest, i.e. the RALC versus CC device effect, investigator and cadaver indicator variables were included in the model as multicollinearity constraints allowed. A *p *value of < 0.05 was regarded as statistically significant.

## Results

The median distance of the stapler from the pelvic floor in the coronal position for the RALC was 1.0 cm (range, 0-4.0 cm), compared to 2.0 cm (0-5.0 cm) for the CC, p = 0.003. In the sagittal position, the median distance from the pelvic floor was 1.6 cm (0-3.5 cm) for the RALC versus 3.3 cm (0-5.0 cm) for the CC, p < 0.0001. (Table 1)

**Table 1:** Distance of stapler from pelvic floor

Stapler placement	Stapler used	Distance (cm) from PF median (range)	Distance (cm) from PF mean (std dev)	Mean Effect	P-value
Coronal	RALC CC	1.0 (0-4.0) 2.0 (0-5.0)	1.4 (1.3) 2.0 (1.2)	-0.54	0.003
Sagittal	RALC CC	1.6 (0-3.5) 3.3 (0-5.0)	1.5 (1.2) 3.2 (1.6)	-1.70	< 0.0001

PF = pelvic floor

RALC = Right Angle Linear Cutter

CC = Curved Cutter

Covariates included investigator and cadaver indicators.

Applied in the coronal position, the RALC was superior to the CC in respect to: 1. the incidence of interference by the symphysis pubis, 2. the number of readjustments of the stapler, 3. ease of placement in the pelvis, 4. impediment of visibility, and 5. access in the pelvis. (Tables 2, 3, 4) Applied in the sagittal position, the RALC was superior to the CC in respect to: 1. the incidence of interference by the symphysis pubis, 2. the number of readjustments of the stapler, 3. ease of placement in the pelvis, 4. ease of placement around the colon and rectum, 5. containment of the entire rectum in the stapler when clamping, 6. ability to hold and retain tissue, 7. visibility rating, and 8. access in the pelvis. (Tables 2, 5, 6) The RALC scored consistently higher than the CC in all questions.

**Table 2:** How many times did you have to readjust the stapler?

Stapler placement	Stapler used	0	1	2	3 or more	Odds Ratio (for fewer times)	P-value
Coronal	RALC CC	18 11	23 20	4 14	2 3	2.75	0.01
Sagittal	RALC CC	19 11	21 17	6 9	1 11	3.53	0.002

RALC = Right Angle Linear Cutter

CC = Curved Cutter

**Table 3:** Stapler Performance: Yes/No questions, Coronal placement

Question	Stapler used	YES	NO	Odds Ratio (for yes)	P Value
Was there interference of the pubic symphysis limiting placement of the device?	RALC CC	1 (2%) 13 (27%)	47 35	0.054	0.006
Was there readjusting of the instrument for optimal placement?	RALC CC	30 (63%) 37 (77%)	18 11	0.47	0.11
Did the instrument impede visibility	RALC CC	1(2%) 23 (48%)	47 25	0.02	0.0003
Was the whole rectum contained in the device after clamping	RALC CC	45 (94%) 46 (96%)	3 2	1.00	0.96

RALC = Right Angle Linear Cutter

CC = Curved Cutter

**Table 4:** Stapler Performance: Rating questions, Coronal placement

Rating	Stapler used	Excellent	Adequate	Poor	Odds Ratio (for poorer results)	P Value
Ease of placement in the pelvis	RALC CC	39 (81%) 21 (45%)	9 (19%) 26 (55%)	0 0	0.18	0.0007
Ease of placement around the colon and rectum	RALC CC	36 (75%) 28 (60%)	11 (23%) 17 (36%)	1 (2%) 2 (4%)	0.43	0.08
Ability to hold and retain tissue without slippage	RALC CC	44 (94%) 45 (96%)	3 (6%) 1 (2%)	0 1 (2%)	1.49	0.67
Visibility	RALC CC	38 (79%) 30 (63%)	10 (21%) 14 (29%)	0 4 (8%)	0.39	0.46
Pelvic access	RALC CC	38 (79%) 18 (38%)	10 (21%) 28 (58%)	0 2 (4%)	0.14	< .0001

RALC = Right Angle Linear Cutter

CC = Curved Cutter

**Table 5:** Stapler Performance: Yes/No questions, Sagittal placement

Question	Stapler used	YES	NO	Odds Ratio (for yes)	P Value
Was there interference of the pubic symphysis limiting placement of the device?	RALC CC	2 (4%) 18 (38%)	46 30	0.064	0.0006
Was there readjusting of the instrument for optimal placement?	RALC CC	29 (60%) 37 (77%)	19 11	0.43	0.07
Did the instrument impede visibility	RALC CC	3 (6%) 6 (13%)	45 42	0.32	0.13
Was the whole rectum contained in the device after clamping	RALC CC	45 (94%) 34 (71%)	3 14	18.08	0.007

RALC = Right Angle Linear Cutter

CC = Curved Cutter

**Table 6:** Stapler Performance: Rating questions, Sagittal placement

Rating	Stapler used	Excellent	Adequate	Poor	Odds Ratio (for poorer results)	P Value
Ease of placement in the pelvis	RALC CC	38 (79%) 13 (27%)	10 (21%) 21 (44%)	0 14 (29%)	0.08	< .0001
Ease of placement around the colon and rectum	RALC CC	36 (75%) 27 (56%)	11 (23%) 8 (17%)	1 (2%) 13 (27%)	0.29	0.007
Ability to hold and retain tissue without slippage	RALC CC	46 (98%) 32 (68%)	0 2 (4%)	1 (2%) 13 (28%)	0.04	0.002
Visibility	RALC CC	38 (79%) 21 (44%)	10 (21%) 14 (29%)	0 13 (27%)	0.16	0.0001
Pelvic access	RALC CC	37 (77%) 15 (31%)	11 (23%) 20 (42%)	0 13 (27%)	0.10	< .0001

RALC = Right Angle Linear Cutter

CC = Curved Cutter

Placed coronally, the stapler rarely obstructed visibility while using the RALC (1/48 applications), while visual impediment was present in 23/48 (48%) using the CC, p = 0.0003. (Table 3) This involved anterior structures, including the prostate, in 95% of the case. Visual impediment was rare with both stapling devices placed sagittally. (Table 5) Superior visibility was experienced with the stapler in the coronal compared to sagittal position with the RALC in 67% of the applications (32/48), and in 96% (43/45) of the applications with the CC. (Table 7) Overall, the surgeons rated the best position for the RALC as coronal in 73% (35/48), and in 98% (44/45) with the CC. (Table 7)

**Table 7:** Preference questions: Coronal and sagittal placement

Question	Stapler used	CORONAL	SAGITTAL
Visually which is best?	RALC CC	32 (67%) 43 (96%)	16 (33%) 2 (4%)
What is the best placement of the device?	RALC CC	35 (73%) 44 (98%)	13 (27%) 1 (2%)

RALC = Right Angle Linear Cutter

CC = Curved Cutter

**Figure 4:**
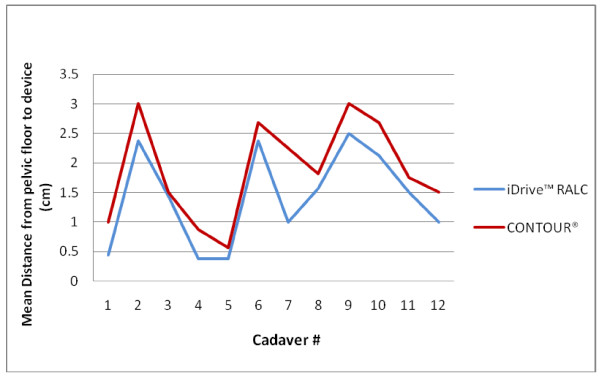
**Mean distance from the pelvic floor to the device using coronal placement**.

**Figure 5:**
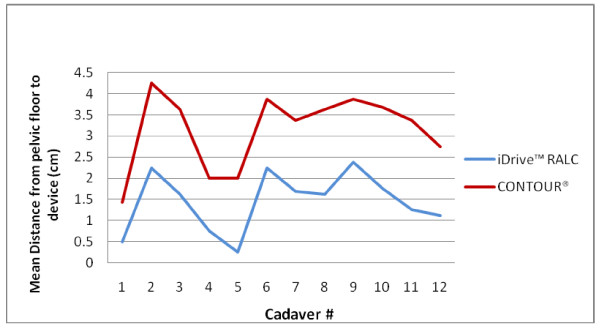
**Mean distance from the pelvic floor to the device using sagittal placement**.

The measurements of pelvic anatomic factors in the twelve cadavers are listed in Table 8. The mean distance from the pelvic floor to the stapling device by cadaver are depicted in Figure 4 and 5, for the coronal and sagittal positions, respectively. Cadavers #2, 3, 6, and 9 demonstrated the longest average stapler height from the pelvic floor, and were deemed the "difficult access" pelvises. Using regression analysis, a longer distance of the stapler from the pelvic floor was found to correlate with a shorter distance between the tip of the coccyx and pubic symphysis (p = 0.002).

**Table 8:** Measurement of anatomic distances

Question	Median* (range)
Umbilicus to pubic symphysis	15 (11-18)
Anterior superior iliac spine (ASIS) to ASIS	24 (20-33)
Pubic symphysis to sacral promontory	12.25 (8-13.5)
Pelvic floor to sacral promontory	12.5 (9-18)
Tip of coccyx to pubis symphysis	11.8 (8-15)
Pelvic inlet**	9.0 (7-13)
Anal verge to pelvic floor	6 (5-10)

Measurements are in cm

*12 cadavers

**Pelvic inlet measured as transverse diameter between right and left pelvic sidewall

## Discussion

Despite recent advances in surgical stapling technology, the development of the ideal stapling device for the distal rectum has been slow. The bony confines of the pelvis, especially android pelvises, cause impediments to visibility and access in the distal rectum. The challenges are more pronounced if the dissected rectum is fatty and bulky, and in the presence of a large rectal tumor. Previous studies have revealed that the male gender, shorter distance of the tumor from the anal verge, and narrower pelvic dimensions are associated with longer operative times and worse outcomes in rectal surgery, including anastomotic leak and positive tumor margins [10-13].

An ideal rectal stapler should be easily advanced to the pelvic floor, have excellent ability to hold tissue, accept a wide range of tissue thickness, seal and divide the rectum reliably and safely, and occlude both the proximal and distal ends of the cut bowel. The challenge in designing such a stapler is that a low-profile stapler may have difficulty incorporating the entire rectum within its jaws, or may require several applications resulting in intersecting staple lines susceptible to ischemia. The rectal transection should ideally be perpendicular to the pelvic floor, but to design a stapler that deflects nearly 90 degrees limits its jaw strength and the deployment of a cutting knife.

In this cadaver study, we compared two stapling devices for the rectum that have similar mechanisms in that they have the ability to occlude both ends of the staple line. This is accomplished by the placement of four rows of staples, with a cutting knife in between the second and third rows. This improves on the traditional method of distal rectal division that involves occluding the bowel distal to the tumor with an atraumatic clamp (to limit spillage of stool and liberated tumor cells), stapling the rectum distal to the clamp, and using a long-handled knife deep in the pelvis to divide the bowel under restricted visual access.

We found that the RALC, compared to the CC, could be placed deeper in the pelvis, has improved visualization, is obstructed less by surrounding tissues, and has pelvic access that was superior (rated "excellent" in 38/48 [79%] as opposed to 18/48 [38%] for the CC). This is likely explained by the smaller profile of the RALC 45-mm single-use load and the perpendicular orientation of the jaws in relation to the shaft of the handle. The RALC Single Use Reload's dimensions are 14.6 mm in width, 40.1 mm in height (closed), and 66.5 mm in length, compared with the CC's dimensions of 25.4 mm in width, 95.0 mm in height (closed), and 62.2 mm in length. We also found that the RALC had excellent and equal access to the deep pelvis whether placed coronally or sagittally, but that the CC was best used coronally. The sagittal placement of the CC resulted in poorer ratings for pelvic access compared with its coronal application. In addition, the CC placed in a sagittal configuration clamped the rectum 1.3 cm more proximally than coronally (3.3 versus 2.0 cm from the pelvic floor).

By performing anatomic measurements in the pelvis, we hoped to evaluate what anatomic factor posed the greatest limitation to distal rectal stapling. A prior study by Gu, et al. that utilized magnetic resonance (MR) pelvimetry in rectal cancer surgery demonstrated that failure of a sphincter-preserving procedure was predicted by a shorter distance from the upper pubic bone to the sacrococcyx, longer distance of the sacrococcyx, and excessive curvature of the sacrum [14]. Another study correlating MR pelvimetry with difficulty of a laparoscopic proctectomy found that a large sagittal pelvic outlet coupled with a narrow transverse intertuberous distance predicted longer operative time [12]. In our current cadaver study, we found that the stapler distance from the pelvic floor was influenced significantly by the distance between the symphysis pubis and the tip of the coccyx. This anterior-posterior restriction of the pelvic outlet seemed to pose more difficulty than any anatomic measurement in the transverse direction or at the pelvic inlet. This knowledge, we hope, could help in the development of improved stapling devices in the future.

There are limitations of this study. First, there is contraction of the mesorectum in a cadaver compared to a live body, and this simplifies placement of the stapling device both around the rectum and into the pelvis. How these stapling devices would perform in the setting of a bulky mesorectum or large rectal tumor remains unanswered by this study. It is possible that the improved access in the deep pelvis with the RALC would be magnified in live surgery; however, it is also possible that the advantages seen in a cadaver model with the RALC will no longer be appreciated in a more technically difficult setting. Secondly, this study only investigated pelvic access and reach. The devices were clamped onto the distal rectum but not fired in order to maximize the number of applications per cadaver. Thus, one critical aspect of stapler performance-how the devices stapled-could not be assessed. Thirdly, investigator bias could not be eliminated, as it was impossible to blind the surgeon to the technique. Furthermore, this research project was funded by one company and not the other, and the participating surgeons received honoraria for their time. Lastly, it would have been interesting to study the influence of body mass index (BMI) on staple placement. This analysis was not performed, however, since the range of the cadavers' BMI (measured by the supplying company) was narrow; the median BMI (and range) of the cadavers was 24.7 (23.7-25.8) kg/m^2^.

## Conclusion

A significant improvement in access and visibility was seen with the RALC compared to the CC in cadavers, and the RALC was the preferred stapler in the majority of applications in the deep pelvis. This could provide important clinical benefit to both the patient and surgeon during difficult rectal surgery.

## Competing interests

Toyooki Sonoda, MD: Received honoraria from Covidien for speaking events, lectures at laparoscopic educational courses, and hourly compensation for work on this cadaver project and manuscript preparation. There is no consultation agreement, stock or stock option ownership, patent-licensing agreements, or research support. There is no financial association with Ethicon.

Juan-Carlos Verdeja, MD: No financial interests, consultant agreements, or speaker bureau agreements with Covidien and Ethicon. Received hourly compensation from Covidien for work on this cadaver project and manuscript review.

David E. Rivadeneira, MD: Received compensation as a consultant and has a consultant agreement with both Covidien and Ethicon. Received honoraria from Covidien for speaking events, laparoscopic educational courses, and hourly compensation for work on this cadaver project and manuscript review. There is no ownership of stocks, stock options, or equity interests, patent-licensing agreements, or research support.

As a group of four participating surgeons, hourly compensation for work on the cadaver laboratory and manuscript preparation totaled $42,900.

## Authors' contributions

TS was involved in the study design, data acquisition, analysis, interpretation of data, and drafting of the manuscript.

JV and DR were involved in the study design, data acquisition, analysis, interpretation of data, and critical revision of the manuscript.

All authors read and approved the final manuscript.
